# Preoperative Prediction of Spread Through Air Spaces in Lung Cancer Using ^18^F-FDG PET–Based Radiomics and Peritumoral Microenvironment Features

**DOI:** 10.3390/diagnostics16050784

**Published:** 2026-03-05

**Authors:** Damla Serçe Unat, Nurşin Agüloğlu, Ömer Selim Unat, Ayşegül Aksu, Bahar Ağaoğlu, Bahattin Dulkadir, Özer Özdemir, Nur Yücel, Kenan Can Ceylan, Gülru Polat

**Affiliations:** 1 Department of Pulmonology, Faculty of Medicine, Izmir Bakircay University, Izmir 35610, Türkiye; 2 Department of Nuclear Medicine, Izmir City Hospital, Izmir 35530, Türkiye; aguloglunursin@gmail.com; 3 Department of Pulmonology, Izmir Dr Suat Seren Chest Disease and Surgery Training and Research Hospital, Health Sciences University, Izmir 35110, Türkiye; unatomerselim@gmail.com (Ö.S.U.); badin.1121@gmail.com (B.D.); ozer_ozdemir@yahoo.com (Ö.Ö.); gulruerbay@yahoo.com (G.P.); 4 Department of Nuclear Medicine, Atatürk Training and Research Hospital, Izmir Katip Celebi University, Izmir 35360, Türkiye; aaysegulgedikli@gmail.com; 5 Department of Thoracic Surgery, Izmir Dr Suat Seren Chest Disease and Surgery Training and Research Hospital, Health Sciences University, Izmir 35110, Türkiye; drbaharagaoglu@hotmail.com (B.A.); kcanceylan@gmail.com (K.C.C.); 6 Department of Pathology, Izmir Dr Suat Seren Chest Disease and Surgery Training and Research Hospital, Health Sciences University, Izmir 35110, Türkiye; n_yucel@yahoo.com

**Keywords:** lung cancer, spread through air spaces (STAS), ^18^F-FDG PET/CT, radiomics, diagnostic accuracy

## Abstract

**Background/Objectives**: Spread through air spaces (STAS) represents an aggressive invasion pattern in lung cancer and is associated with unfavorable oncologic outcomes. As STAS is currently identifiable only on postoperative pathology, reliable preoperative, noninvasive prediction remains a clinical challenge. This study aimed to evaluate the feasibility of predicting STAS using ^18^F-fluorodeoxyglucose positron emission tomography/computed tomography (^18^F-FDG PET/CT)-derived radiomic and clinicoradiomic models. **Methods**: In this retrospective study, patients who underwent surgical resection for lung cancer with available preoperative ^18^F-FDG PET/CT imaging were analyzed. Radiomic features were extracted from intratumoral and peritumoral regions. Clinical, radiomic-only, and combined clinicoradiomic models were developed using LASSO-based feature selection and multivariable logistic regression. Model performance was evaluated using nested cross-validation, receiver operating characteristic analysis, calibration assessment, and decision curve analysis. **Results**: Radiomic features reflecting intratumoral metabolic characteristics and peritumoral tissue heterogeneity were significantly associated with STAS. The combined clinicoradiomic model demonstrated superior discriminative performance compared with the clinical and radiomic-only models (mean AUC ≈ 0.75), along with favorable calibration (Brier score = 0.20) and improved clinical net benefit across relevant threshold probabilities. Lower eosinophil count, lower SUVmin_tumor, and lower intratumoral SUV skewness emerged as independent predictors of STAS. **Conclusions**: Preoperative prediction of STAS in lung cancer is feasible using PET/CT-based radiomic analysis integrating intratumoral, peritumoral, and clinical features. This noninvasive approach provides biologically relevant information beyond conventional anatomical assessment and warrants further validation in prospective, multicenter cohorts.

## 1. Introduction

Lung cancer remains one of the leading causes of cancer-related mortality worldwide [1]. Despite significant advances in surgical techniques, targeted therapies, and immunotherapies in recent years, patient prognosis largely depends on accurate staging and a deeper understanding of tumor biology [2]. In this context, there is a growing need for novel prognostic markers that can more precisely predict the clinical course of the disease, beyond the conventional (Tumor-Node-Metastasis) TNM staging system [3].

Prognostic factors identifiable in the preoperative period are central to personalized treatment strategies, especially for surgical candidate selection and resection planning. Consequently, the identification of novel markers reflecting invasion patterns and tumor biological behavior has become an important focus of contemporary lung cancer research.

In this context, invasion-related features that reflect the interaction between the tumor and the surrounding lung parenchyma, as well as patterns of tumor spread along air spaces, have emerged as a new paradigm for characterizing tumor biology and refining prognostic assessment. Although tumor invasion through air spaces has long been recognized in lung cancer, it was formally defined as a distinct invasion mechanism by the World Health Organization (WHO) in 2015 under the term “tumor spread through air spaces (STAS).” The presence of STAS has been shown to be associated with worse survival outcomes and has been proposed as an independent prognostic factor in lung adenocarcinomas across all stages, as well as in other histological subtypes of lung cancer [4].

Numerous clinical studies published in recent years have demonstrated that the presence of STAS is associated with poorer prognosis, an increased risk of local recurrence, and unfavorable survival outcomes in lung cancer [5,6,7,8]. STAS has been reported to represent an invasion pattern reflecting the biological aggressiveness of the disease independent of tumor stage and may be considered an independent prognostic factor [5,6]. The presence of STAS has been shown to confer more aggressive tumor behavior, with important implications for clinical decision-making, including the determination of the extent of surgical resection and postoperative treatment planning. STAS positivity has been associated with an increased risk of local recurrence following sublobar resection, even in patients with early-stage disease, prompting consideration of more extensive resection strategies or the need for adjuvant therapy [7,9]. Accordingly, STAS is increasingly regarded not only as a prognostic marker but also as a potential clinical factor that may influence treatment decisions [6,7].

In recent years, radiomic approaches enabling the quantitative analysis of high-dimensional data derived from medical imaging have been increasingly applied to a wide range of oncologic diseases, particularly lung cancer, with the aim of improving diagnostic accuracy, predicting prognosis, and estimating treatment response [10,11,12]. Clinicoradiomic models developed through the integration of clinical variables with radiomic features have demonstrated superior predictive performance compared with imaging or clinical parameters alone, allowing a more comprehensive representation of tumor biological behavior [13,14]. These advances offer substantial potential, particularly for preoperative risk stratification and personalized treatment planning.

Another emerging area of interest in lung cancer research in recent years is the role of the tumor microenvironment in shaping disease behavior. Accumulating evidence suggests that the composition of the tumor microenvironment, including immune cell infiltration and interactions with local and intratumoral microbiota, plays a critical role in tumor progression, treatment outcomes, and response to immunotherapy. The dynamic balance between immunosuppressive and immunostimulatory components within this microenvironment may influence clinical behavior and has been proposed to contribute to histopathological features associated with invasive growth patterns, such as STAS. Accordingly, a deeper understanding of these microenvironmental interactions is considered essential for the development of predictive biomarkers and personalized therapeutic strategies in lung cancer [15].

In this context, artificial-intelligence- and deep-learning-based approaches have recently been explored for the recognition and quantification of STAS. In a study published in 2024, Feng et al. reported that a deep learning model developed using digital pathology data demonstrated promising performance in the detection of STAS [16].

Although radiomic and artificial intelligence–based methods have shown encouraging results for STAS identification, the role of imaging-derived features reflecting the tumor microenvironment in the preoperative, noninvasive prediction of STAS remains insufficiently characterized. In particular, the contribution of clinicoradiomic models that integrate radiomic features with clinical variables to STAS-related risk stratification has been addressed in only a limited number of studies. Given the prognostic and potential treatment-guiding implications of STAS, there is a clear need for preoperative, noninvasive predictive models incorporating microenvironment-related radiomic features.

Accordingly, the aim of this study was to preoperatively predict the presence of STAS in patients with lung cancer using PET/CT-based radiomic and tumor microenvironment analyses, and to compare the diagnostic performance of radiomic-only and clinicoradiomic models.

## 2. Materials and Methods

### 2.1. Study Design and Patient Population

This retrospective study included 1295 patients who underwent surgical resection for lung cancer between 2018 and 2024. Among these, 65 patients were pathologically diagnosed with STAS. Preoperative PET/CT imaging was available in 47 STAS-positive patients, who constituted the STAS-positive group.

Of the remaining 1230 patients, those with T4 disease (*n* = 89), pathological N2 involvement (*n* = 250), small cell lung cancer (*n* = 95), or unknown STAS status (*n* = 272) were excluded. Following these exclusions, 524 patients were eligible for control selection.

Only patients with histologically confirmed non-small-cell lung cancer were included. The cohort comprised multiple histological subtypes, including adenocarcinoma and squamous cell carcinoma. Detailed histopathological information, including histological subtype and tumor differentiation grade, was available for all patients.

Patients who received neoadjuvant chemotherapy, radiotherapy, or immunotherapy prior to surgery were excluded to avoid treatment-related alterations in tumor metabolic and radiomic characteristics. All included patients underwent upfront surgical resection with curative intent.

A frequency-matched sampling strategy was applied to achieve comparable distributions of T stage and tumor size between STAS-positive and STAS-negative groups. Control patients were selected to achieve a distribution of T stage and tumor size comparable to that of the STAS-positive group. First, the distribution of T stage and tumor size among STAS-positive patients was determined. Subsequently, STAS-negative patients were randomly selected using an independent computer-based software to match this distribution.

Initially, 104 STAS-negative patients with a comparable T-stage distribution were identified. However, due to the availability of preoperative PET/CT imaging, 52 STAS-negative patients were ultimately included as the control group. The final study cohort consisted of 99 patients (47 STAS-positive and 52 STAS-negative), all of whom had available preoperative PET/CT imaging and comparable T stage and tumor size.

Ethical approval was obtained from the Institutional Review Board (Approval No: 2025/33-26), and the requirement for written informed consent was waived due to the retrospective use of anonymized data.

### 2.2. Clinical Data Collection

Clinical data were retrospectively obtained from electronic medical records. Demographic variables, including age and sex, and clinical characteristics such as smoking status, tumor location, and type of surgical resection were recorded. Pathological staging was determined according to the TNM classification system in use at the time of diagnosis. 

For model development, clinicopathological variables considered clinically relevant or previously reported to be associated with prognosis were included in the analysis. Pathological assessment was performed independently of imaging analyses, and investigators involved in radiomic feature extraction and model construction were blinded to pathological outcomes.

### 2.3. PET/CT Imaging Protocol

All patients underwent preoperative ^18^F-fluorodeoxyglucose positron emission tomography/computed tomography (^18^F-FDG PET/CT) imaging using a Philips Gemini TF 16-slice combined PET/CT scanner (Philips Medical Systems, Cleveland, OH, USA). The same PET/CT system was used for all patients to ensure imaging consistency. Following a minimum of 6 h of fasting (blood glucose levels < 150 mg/dL), 8–15 mCi of ^18^F-FDG (approximately 2.5 MBq/kg body weight) was administered intravenously in accordance with the European Association of Nuclear Medicine (EANM) guidelines, version 2.0 [17]. Image acquisition was initiated 60 ± 5 min after tracer injection.

Non-contrast-enhanced CT images were first acquired using the following parameters: 140 kV, 100 mAs, and 5 mm slice thickness. Subsequently, PET images were obtained via whole-body scanning from the skull apex to the proximal thighs, with an emission acquisition time of 1.5 min per bed position and a total of 9 or 10 bed positions. Attenuation correction was performed using the acquired CT images. The voxel size was 4 × 4 × 4 mm. PET images were reconstructed using the row-action maximum likelihood algorithm (RAMLA).

### 2.4. ^18^F-FDG PET/CT Texture-Volumetric Analysis

^18^F-FDG PET/CT images were analyzed using LIFEx software LIFEx, Orsay, France (https://www.lifexsoft.org, accessed 10 January 2026) [18]. PET/CT images in DICOM format were transferred to the software for analysis. For each lesion, an initial volume of interest (VOI) was created to ensure accurate tumor delineation while excluding adjacent physiological uptake. Segmentation was performed by an experienced nuclear medicine physician specializing in thoracic PET/CT imaging, who was blinded to pathological findings, including STAS status.

**Figure 1:**
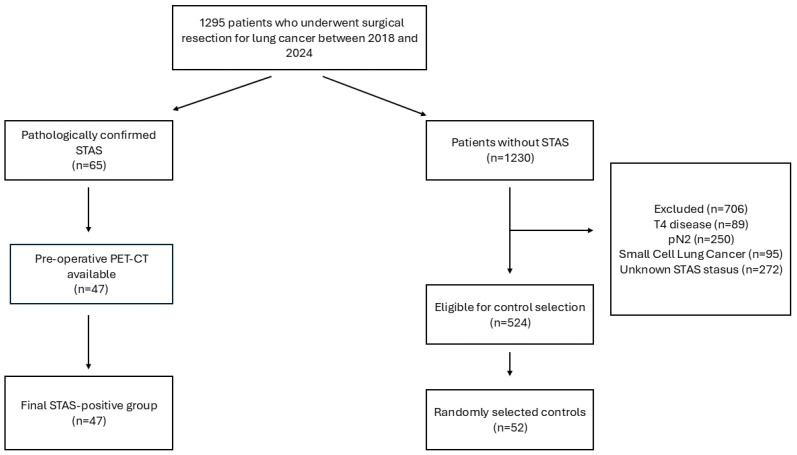
Flow diagram of patient selection. Among 1295 patients who underwent surgical resection for lung cancer, 65 were pathologically diagnosed with STAS. Forty-seven STAS-positive patients with available preoperative PET/CT constituted the study group. After applying exclusion criteria to the remaining patients, 524 were eligible for control selection.

The intratumoral VOI was defined as the metabolically active tumor volume. The region of interest of the target lesion was semi-automatically delineated on hybrid ^18^F-FDG PET/CT images using a 41% SUVmax threshold by a nuclear medicine physician with 10 years of experience in PET/CT imaging (Figure 1). SUV normalized to body weight (SUVbw) was used as the SUV type. Conventional SUV parameters, histogram-based features, and shape parameters were extracted. Textural features were subsequently derived using gray-level co-occurrence matrix (GLCM), gray-level run length matrix (GLRLM), neighborhood gray-level difference matrix (NGLDM), and gray-level zone length matrix (GLZLM) analyses. These features are summarized in Table 1.

Areas with SUV values above 2.5 on whole-body images were automatically segmented. An initial VOI was generated for each lesion and manually adjusted when necessary to ensure accurate tumor delineation while excluding adjacent physiological uptake. The obtained VOIs were evaluated individually, and SUVmean and metabolic tumor volume (MTV) values were calculated for regions corresponding to the primary tumor. A 41% SUVmax threshold was applied to the VOIs [17,19]. Total lesion glycolysis (TLG) was calculated by multiplying MTV and SUVmean. CT images were used solely for attenuation correction, and CT-derived features were not included in texture analysis.

Texture matrices were computed using 64 gray levels to resample the region of interest. Absolute resampling was performed using 64 bins with SUV values ranging from a minimum of 0 to a maximum of 50, based on the maximum SUV values observed in the study cohort.

### 2.5. Tumor Microenvironment–Related Radiomic Analysis

To assess tumor microenvironment–related imaging characteristics, a peritumoral VOI was generated by isotropically expanding the intratumoral VOI by 7 mm in all directions. This expansion distance was predefined and selected in accordance with previous radiomics studies evaluating peritumoral tumor microenvironment features, where margins in the range of 5–10 mm have been commonly used.

Non-pulmonary structures and areas of physiological FDG uptake, including the mediastinum, heart, major vessels, chest wall, and central airways, were carefully excluded. The peritumoral VOI was restricted to the lung parenchyma to reflect tumor–host interactions within the surrounding tissue. All peritumoral regions were manually reviewed and adjusted when necessary to avoid inclusion of adjacent vessels, bronchi, air spaces, or other non-lung tissues. The peritumoral VOIs were visually inspected and manually adjusted to prevent overlap with adjacent vessels, bronchi, and other non-lung structures.

Additional radiomic features were extracted from the peritumoral region identified during the segmentation process to evaluate imaging characteristics related to the tumor microenvironment. Radiomic features derived from the peritumoral region were used to characterize metabolic heterogeneity and spatial patterns potentially reflecting tumor–host interactions beyond the intratumoral compartment. These tumor microenvironment–related features were analyzed both separately and in combination with intratumoral radiomic features to explore their complementary predictive value.

**Figure 2:**
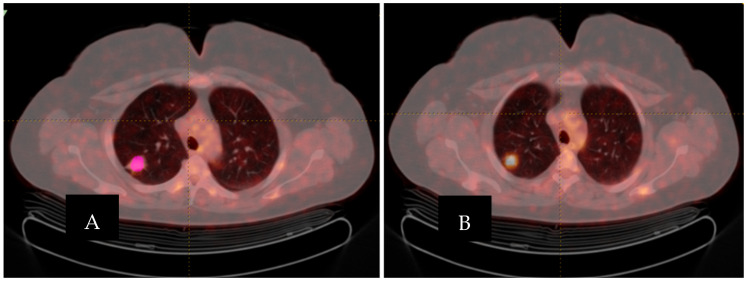
(**A**). PET/CT fusion image, (**B**). Segmentation (with pink color) of the lung lesion in the ^18^F-FDG PET/CT with LIFEx software.

Both intratumoral and tumor microenvironment–related radiomic features were subsequently incorporated into radiomic-only and clinicoradiomic models for the preoperative prediction of STAS (Figure 2).

### 2.6. Surgical Evaluation and Procedure

All patients underwent surgical resection with curative intent in accordance with current international guidelines for lung cancer management. The surgical approach and extent of resection were determined based on tumor characteristics, pulmonary reserve, comorbidities, and surgeon preference. Anatomical resections, including lobectomy or segmentectomy, were performed via video-assisted thoracoscopic surgery (VATS) or open thoracotomy, while sublobar resection was reserved for selected patients with limited cardiopulmonary reserve or small peripheral tumors. Wedge resection was performed only when anatomical resection was not feasible, and conversion from VATS to open thoracotomy was undertaken when required for oncological or technical reasons.

Systematic mediastinal lymph node dissection or sampling, including hilar and mediastinal nodal stations, was routinely performed according to institutional practice and international staging recommendations. Resected specimens were immediately sent for pathological examination, with tumor size measured on fresh specimens and resection margins assessed to confirm complete (R0) resection.

Postoperative management followed standardized institutional protocols. Perioperative complications, length of hospital stay, and postoperative outcomes were recorded, and surgical and pathological data were prospectively collected and retrospectively reviewed for analysis.

### 2.7. Histopathological Evaluation

Histopathological evaluation was performed on surgically resected specimens using routine hematoxylin–eosin-stained sections by experienced thoracic pathologists as part of standard clinical practice. Tumor histological subtypes were classified according to the WHO classification of lung tumors. The presence or absence of STAS was assessed based on final pathology reports and recorded as a binary variable (STAS-positive or STAS-negative). STAS was defined as the presence of tumor cells beyond the edge of the main tumor mass, identified as micropapillary structures, solid tumor nests, or single tumor cells within the surrounding alveolar spaces at least one alveolar space away from the primary tumor. Pathological assessment of STAS was performed independently and subsequently confirmed, with pathologists blinded to clinical data, nuclear medicine findings, and radiomic analyses.

### 2.8. Model Construction

Two predictive models were developed for the preoperative prediction of STAS. The first was a radiomic-only model, incorporating selected intratumoral and peritumoral radiomic features derived from preoperative PET/CT imaging. The second was a combined clinicoradiomic model integrating radiomic features with clinically relevant variables available prior to surgery.

All variables used for model development were restricted to preoperative clinical, laboratory, and imaging data. Postoperative or pathological variables were not used as model inputs.

Before model construction, feature dimensionality was reduced to minimize redundancy and prevent overfitting. Feature selection was performed using a regularization-based approach, and highly correlated or unstable features were excluded. The selected features were subsequently entered into multivariable model development.

Model construction was carried out using supervised classification methods. Radiomic-only and clinicoradiomic models were developed separately to enable direct comparison of their predictive performance and to assess the incremental value of integrating radiomic features with preoperative clinical parameters.

### 2.9. Radiomic Feature Extraction and Feature Selection

Clinical and radiomic variables were evaluated as candidate predictors using univariate screening, and feature selection was subsequently performed using least absolute shrinkage and selection operator (LASSO) with internal cross-validation to reduce dimensionality and minimize multicollinearity.

In accordance with TRIPOD recommendations, variables retained after LASSO selection were subsequently entered into multivariable logistic regression, and model parameters were reported as odds ratios (ORs) with 95% confidence intervals.

The final feature set included inflammatory markers (eosinophil and neutrophil counts), intratumoral PET parameters (SUVmin_tumor and SUVSkewness_tumor), and texture features reflecting intra- and peritumoral heterogeneity (GLRLM_LRLGE_tumor, NGLDM_Coarseness_tumor, GLZLM_LZLGE_tumor, and peritumoral NGLDM_Coarseness).

Image preprocessing and feature extraction were performed using LIFEx software, which applies standardized radiomics calculation procedures consistent with IBSI recommendations. Texture features were computed using fixed voxel size and absolute resampling with 64 gray levels, ensuring standardized discretization across all cases.

### 2.10. Model Validation and Performance Evaluation

Model performance was assessed using a nested cross-validation strategy to minimize information leakage. In the outer loop, five-fold cross-validation was applied for unbiased performance estimation. In each outer training fold, an inner cross-validation loop was used for all data-driven procedures, including univariate feature screening, feature standardization, LASSO-based feature selection, and hyperparameter optimization.

Features and model coefficients selected in the inner loop were subsequently applied to the held-out outer test fold. All performance metrics, including AUC, sensitivity, specificity, Youden-derived cutoff, Brier score, calibration curves, and decision curve analysis, were calculated exclusively using out-of-fold predictions from the outer loop.

Predictive performance was quantified using receiver operating characteristic (ROC) curve analysis. Model calibration was evaluated using calibration plots and Brier scores, and clinical utility was assessed using decision curve analysis.

Apparent (training-set) performance was not reported. This validation framework ensured unbiased estimation of model generalizability and minimized optimistic bias.

### 2.11. Statistical Analysis

Continuous variables were presented as mean ± standard deviation or median (interquartile range), according to their distribution. Between-group comparisons were performed using Student’s *t* test or the Mann–Whitney U test, as appropriate, while categorical variables were compared using Fisher’s exact test.

For STAS prediction, a hierarchical logistic regression modeling strategy was applied. The clinical model included eosinophil count and neutrophil count. The clinical–tumoral model was constructed by adding SUVmin_tumor, and the combined model was obtained by further incorporating SUVSkewness_tumor and peritumoral NGLDM-derived Coarseness.

All continuous variables were standardized using Z-score transformation prior to analysis. Multicollinearity was assessed using variance inflation factors, with values below 5 considered acceptable. The event-per-variable ratio was maintained above recommended thresholds. The event-per-variable (EPV) ratio was calculated based on 47 STAS-positive events and six predictors included in the final combined model, yielding an EPV of approximately 7.8, which is considered acceptable for multivariable logistic regression.

Model discrimination was evaluated using ROC curve analysis based on out-of-fold predictions from nested cross-validation. AUC values were summarized as mean ± standard deviation across outer folds.

Model calibration was assessed using Brier scores and calibration plots. Clinical utility was evaluated using decision curve analysis. Optimal classification thresholds were determined based on cross-validated Youden indices derived from out-of-fold predictions.

All statistical analyses were performed using R software (version 4.3.3). Feature selection and dimensionality reduction were conducted using the glmnet package, ROC analyses were performed using the pROC package, internal validation procedures were carried out using the caret package, clinical utility was assessed using the dcurves package, descriptive statistics were generated using the tableone package, and graphical visualizations were created using ggplot2. The study was conducted and reported in accordance with the TRIPOD (Transparent Reporting of a Multivariable Prediction Model for Individual Prognosis or Diagnosis) guidelines.

## 3. Results

### 3.1. Patient Characteristics and Univariate Analyses

Among the total of 99 patients, STAS positivity was identified in 47.5% (*n* = 47). The mean age of the entire cohort was 66.4 ± 8.65 years, and 25.3% of patients were female. A history of current or former smoking was present in 61.7% of patients. Centrally located tumors accounted for 24.2% of the cohort. Median laboratory values were as follows: Lactate Dehydrogenase (LDH) 177 U/L (160–198.5), C-Reactive Protein (CRP) 3.65 mg/L (1.72–10.15), neutrophil count 4700/mm^3^ (3605–6705), eosinophil count 160/mm^3^ (100–220), and neutrophil-to-lymphocyte ratio (NLR) 2.14 (1.70–3.35).

The pathological stage distribution of the cohort was as follows: Stage I: 49.5%, Stage II: 29.3%, and Stage III: 21.2%.

No significant differences were observed between STAS-positive and STAS-negative groups with respect to age, sex, or smoking history (all *p* > 0.05). STAS was significantly more frequent in peripherally located tumors compared with centrally located tumors (87.2% vs. 65.4%, *p* = 0.018).

In laboratory analyses, LDH levels were significantly higher in the STAS-positive group (median 182 vs. 170.5 U/L, *p* = 0.027), whereas neutrophil count (*p* = 0.005), eosinophil count (*p* = 0.036), and CRP level (*p* = 0.005) were significantly lower.

Regarding radiomic parameters, SUVmin_tumor values were significantly lower in STAS-positive patients compared with STAS-negative patients (median 1.41 [IQR: 0.96–1.88] vs. 2.39 [IQR: 1.74–3.91], *p* < 0.001). Conversely, peritumoral NGLDM_Coarseness values were significantly higher in the STAS-positive group (median 0.0101 [IQR: 0.0069–0.0161] vs. 0.0076 [IQR: 0.0045–0.0109], *p* = 0.006), indicating increased peritumoral structural heterogeneity.

In univariate logistic regression analysis, central tumor location (OR = 0.276, *p* = 0.014), eosinophil count (OR = 0.995, *p* = 0.023), and SUVmin_tumor (OR = 0.55, *p* < 0.001) were significantly associated with STAS. Peritumoral NGLDM_Coarseness showed a markedly elevated odds ratio with wide confidence intervals, reflecting numerical instability related to its distributional properties; therefore, this variable was further stabilized through standardization in subsequent multivariable analyses. In addition to SUVmin_tumor and peritumoral NGLDM_Coarseness, several texture-derived features were evaluated in univariate analyses. SUVSkewness_tumor demonstrated a significant association with STAS in logistic regression analysis (OR 0.34, 95% CI 0.12–0.96, *p* = 0.041), although only a trend was observed in non-parametric testing (*p* = 0.070). NGLDM_Coarseness_tumor and NGLDM_Coarseness_peritumor were also significantly associated with STAS; however, these parameters exhibited extremely large odds ratios and wide confidence intervals, indicating numerical instability. Other radiomic features, including GLRLM_LRLGE_tumor and GLZLM_LZLGE_tumor, did not show significant associations with STAS (all *p* > 0.05). Full regression results for the radiomic-only and combined clinicoradiomic models are presented in Table 2 and Table 3.

### 3.2. Multivariable Model Performance and Internal Validation

Nested cross-validation demonstrated progressive improvement in predictive performance across models. The combined clinicoradiomic model achieved the highest discrimination (mean AUC = 0.75 ± 0.04), followed by the radiomic-only model (AUC = 0.69 ± 0.11) and the clinical model (AUC = 0.66 ± 0.13).

The combined model showed superior stability, reflected by its lower variability across folds. At the optimal cross-validated cutoff value, the combined model achieved an overall accuracy of 65.7%, with sensitivity of 68.1% and specificity of 63.5%.

### 3.3. Model Comparisons and Incremental Contribution Analysis

Likelihood ratio testing demonstrated that the addition of SUVmin_tumor significantly improved model fit over the clinical model. Further incorporation of peritumoral texture features resulted in additional performance gains.

Decision curve analysis based on out-of-fold predictions showed that the combined model consistently provided greater net clinical benefit across clinically relevant threshold probabilities compared with the clinical and radiomic-only models. These findings support the incremental value of integrating metabolic and microenvironment-related radiomic features into STAS prediction.

### 3.4. Multivariable Risk Estimates and Calibration

In the final combined model, independent predictors of STAS were lower eosinophil count (OR 0.993 per cell/mm^3^, 95% CI 0.987–0.999, *p* = 0.024), lower SUVmin_tumor (SUVmin_tumor; OR 0.624, 95% CI 0.413–0.941, *p* = 0.025), and lower intratumoral SUV skewness (OR 0.174, 95% CI 0.042–0.723, *p* = 0.016). Additional radiomic texture features selected during LASSO (GLRLM_LRLGE_tumor, NGLDM_Coarseness_tumor, and GLZLM_LZLGE_tumor) were not independently associated with STAS in multivariable analysis (all *p* > 0.05). Peritumoral NGLDM_Coarseness showed a trend toward increased risk but with very wide confidence intervals, suggesting numerical instability related to feature scaling and distribution (OR 7.61 × 10^16^, *p* = 0.399).

**Figure 3:**
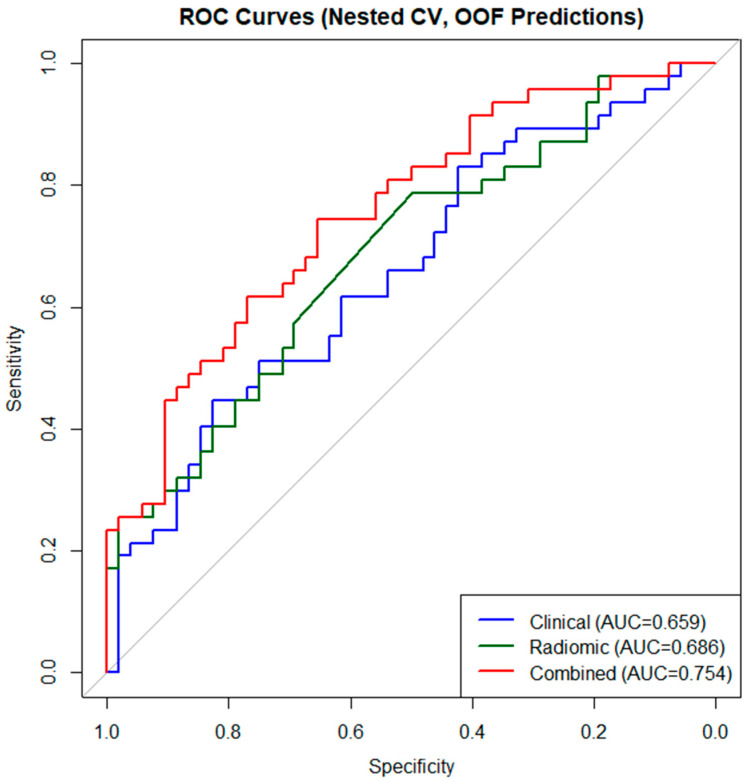
**ROC Curves Based on Nested Cross-Validation for the Prediction of STAS.** This figure presents the ROC curves for the clinical, radiomic-only, and combined clinicoradiomic models using out-of-fold predictions derived from nested cross-validation. The *x*-axis represents 1−specificity (false positive rate), and the *y*-axis represents sensitivity (true positive rate). The dashed diagonal line indicates the performance of a random classifier (AUC = 0.5). The combined clinicoradiomic model demonstrates superior discriminative performance compared with the clinical and radiomic-only models, reflecting the incremental value of integrating radiomic features with clinical parameters for preoperative STAS prediction.

**Figure 4:**
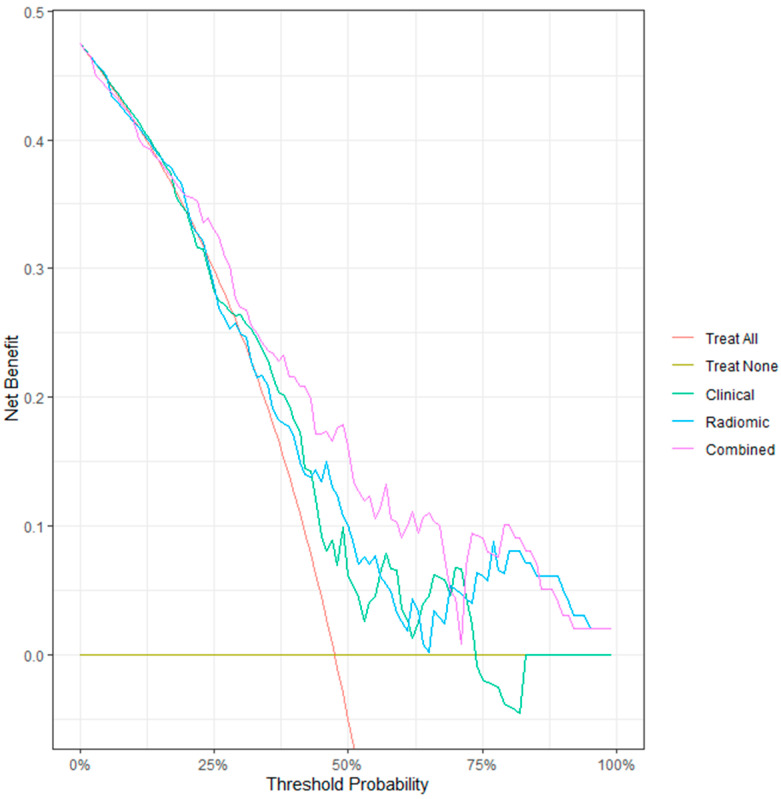
**Decision Curve Analysis Based on Nested Cross-Validation for Predicting STAS.** Decision curve analysis using out-of-fold predictions from nested cross-validation compares the net clinical benefit of the clinical, radiomic-only, and combined clinicoradiomic models across a range of threshold probabilities. The combined model consistently demonstrates the highest net benefit over most clinically relevant thresholds, indicating superior potential for guiding preoperative decision-making compared with either clinical or radiomic features alone.

**Figure 5:**
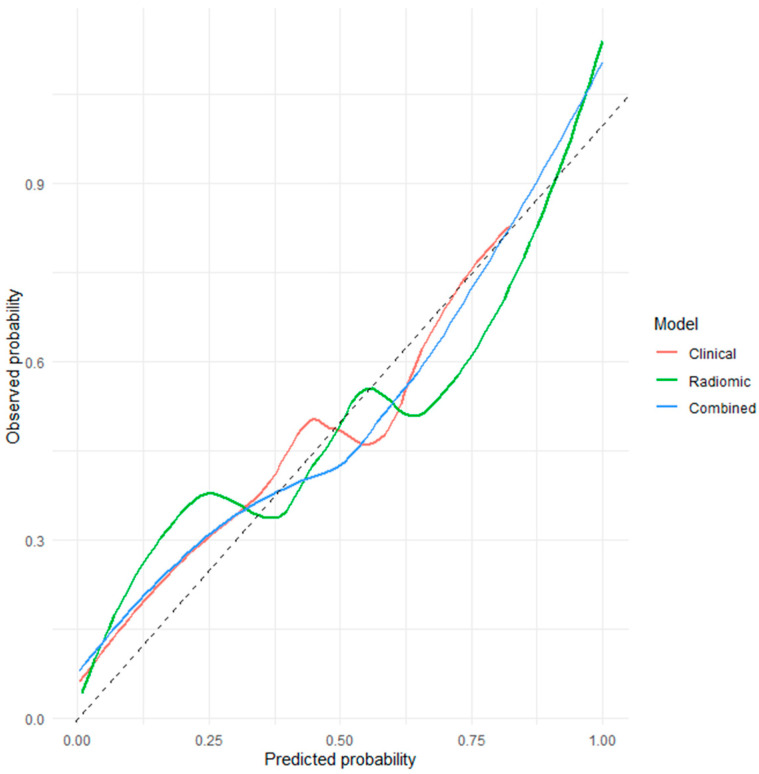
**Calibration Plots Based on Out-of-Fold Predictions for STAS Prediction Models.** Calibration curves derived from nested cross-validation assess the agreement between predicted probabilities and observed STAS frequencies for the clinical, radiomic-only, and combined models. The combined model shows the closest alignment with the ideal reference line, indicating superior calibration performance. This finding is further supported by its lower Brier score, reflecting improved reliability of risk estimation.

Calibration assessed on out-of-fold predictions demonstrated good agreement between predicted and observed risks. The combined model achieved the lowest Brier score (0.202), indicating superior overall calibration compared with the clinical and radiomic-only models. Because the cohort was assembled using frequency-matched sampling, predicted probabilities should not be interpreted as absolute population risk estimates (Figure 3, Figure 4 and Figure 5). Detailed TRIPOD-compliant coefficient tables for the final combined and radiomic models are provided in the Supplementary Materials (Tables S1 and S2).

## 4. Discussion

In this study, ^18^F-FDG PET/CT–based radiomic and clinicoradiomic models were developed and internally validated for the preoperative prediction of STAS in patients with non-small-cell lung cancer. STAS, which is conventionally assessed through histopathological examination of resected specimens, was analyzed in relation to clinical, laboratory, and radiomic features in a surgically treated cohort.

STAS was associated with peripheral tumor location and selected metabolic and radiomic features. Preoperative identification of patients at high risk for STAS may have important implications for surgical planning. Therefore, patients with a high predicted probability of STAS may be considered for lobectomy rather than sublobar resection to achieve better oncologic outcomes. These findings are supported by imaging parameters and radiomic features reflecting peritumoral tissue heterogeneity. The inclusion of radiomic variables alongside clinical parameters resulted in improved model performance compared with clinical models alone. Differences in net benefit across a range of threshold probabilities were observed between the evaluated models in decision curve analysis. The combined clinicoradiomic model demonstrated acceptable calibration, indicating agreement between predicted probabilities and observed STAS status within the study population.

The prognostic significance of STAS was first systematically described by Kadota and colleagues, who demonstrated its association with worse survival and higher recurrence rates in lung adenocarcinoma [5]. Subsequently, Warth et al. confirmed that STAS constitutes an independent prognostic factor across broader patient populations [6]. Shiono and Yanagawa further highlighted the adverse prognostic impact of STAS in stage I disease, emphasizing its prognostic value beyond conventional staging systems [8]. Similarly, Toyokawa et al. reported inferior oncological outcomes in patients with STAS-positive tumors, reinforcing the clinical importance of this invasion pattern [9]. Collectively, these studies establish STAS as more than a histopathological observation, positioning it as a surrogate marker of aggressive tumor behavior.

From a clinical perspective, STAS challenges the adequacy of anatomy-based staging systems alone. Although TNM classification remains the cornerstone of lung cancer management [2], it does not capture microscopic invasion patterns or tumor–host interactions. Evidence demonstrating significantly worse outcomes in STAS-positive tumors of similar stage and size suggests that a subset of anatomically early-stage tumors may behave biologically aggressively [6,8,9]. Preoperative identification of STAS could therefore facilitate a shift from purely anatomy-driven decision-making toward a more biology-oriented treatment strategy.

Radiomic analysis enables the extraction of quantitative descriptors that characterize spatial heterogeneity within medical images and has increasingly been applied to explore both tumor-intrinsic properties and the surrounding tissue context [10,11,12]. Although STAS represents a distinct histopathological invasion pattern, several CT-based radiomic studies have demonstrated that intratumoral heterogeneity features are associated with STAS and can achieve meaningful predictive performance [20,21]; however, PET/CT-based radiomic approaches specifically targeting STAS remain scarce. In this context, the present study extends prior work by jointly evaluating intratumoral metabolic features and peritumoral radiomic characteristics derived from ^18^F-FDG PET/CT, thereby capturing complementary information related to both tumor structure and the adjacent microenvironment. The inclusion of the peritumoral NGLDM-derived coarseness feature highlights the potential of radiomic texture metrics to reflect metabolic and structural heterogeneity in lung parenchyma surrounding the tumor, an area closely linked to the biological processes underlying STAS. 

A major contribution of the present study is the integration of peritumoral, microenvironment-related radiomic features derived from ^18^F-FDG PET/CT into STAS prediction models. Although STAS is defined histopathologically by tumor cell spread beyond the main tumor edge into surrounding alveolar spaces, metabolic and structural alterations in the adjacent lung parenchyma represent biologically plausible imaging correlates of this invasion pattern [4]. Similar peritumoral radiomic features, including NGLDM coarseness, have previously been associated with microenvironmental heterogeneity and tumor–host interactions in oncologic PET/CT imaging studies [22]. Within this framework, the contribution of the peritumoral NGLDM-derived coarseness feature in our models suggests that imaging-based texture metrics may capture aspects of microenvironmental disruption related to invasive behavior, providing complementary information beyond intratumoral features alone while maintaining a noninvasive approach. Recent spatial transcriptomic analyses have further demonstrated that tumor cells involved in STAS exhibit distinct molecular and microenvironmental profiles at the tumor–airspace interface compared with the main tumor mass, supporting the biological plausibility of peritumoral heterogeneity captured by imaging-based approaches [23]. This finding aligns with previous oncologic imaging literature indicating that peritumoral radiomic features may carry prognostic relevance across different tumor types [24].

Among intratumoral radiomic parameters, SUVmin_tumor emerged as an independent and robust predictor of STAS. Previous PET-based radiomic studies have suggested that parameters reflecting metabolic heterogeneity and spatial distribution may convey more biologically meaningful information than single-voxel intensity metrics such as SUVmax [25,26]. Low-uptake regions within tumors have been associated with hypoxia, necrosis, and irregular invasive growth patterns. Our findings extend this concept to STAS, indicating that intratumoral metabolic heterogeneity captured by SUVmin may reflect underlying invasive potential.

From a surgical perspective, the ability to estimate STAS risk preoperatively carries important clinical implications. Radiomics-based preoperative risk assessment may complement intraoperative decision-making. Frozen-section evaluation for STAS has shown variable sensitivity and may not always reliably detect this feature. A noninvasive imaging-based risk stratification model could provide valuable preoperative information and support surgical strategies, particularly in borderline cases or when frozen-section results are inconclusive. Prior studies have demonstrated higher local recurrence rates following sublobar resection in STAS-positive tumors, whereas lobectomy has been associated with improved oncologic outcomes in this subgroup [7,8]. Although the present study does not aim to directly guide surgical decision-making, it provides a noninvasive tool to estimate the biological risk underlying surgical strategies, potentially supporting more individualized operative planning.

More broadly, our findings reflect an ongoing paradigm shift in lung cancer management. Traditional reactive approaches based on postoperative pathology are increasingly being complemented by proactive strategies that aim to anticipate tumor behavior before treatment. In this context, PET/CT radiomics and microenvironment analysis should be viewed not merely as advanced imaging techniques, but as biologically informative windows into tumor aggressiveness. Emerging evidence from digital pathology and deep learning-based STAS detection further supports this integrative, biology-driven framework [16].

Although decision curve analysis demonstrated a higher net benefit for the combined model across a wide range of threshold probabilities, these findings should be interpreted with caution. Because the study cohort was constructed using frequency-matched sampling rather than reflecting population-level prevalence, the predicted probabilities may not represent absolute risk estimates. Therefore, decision curve analysis was used to compare the relative clinical utility of the models within the study cohort, and model performance was primarily interpreted in terms of discrimination.

Several limitations should be acknowledged. The retrospective, single-center design may limit generalizability. The study cohort was assembled using frequency-matched sampling rather than reflecting population-level STAS prevalence; therefore, predicted probabilities derived from the model should not be interpreted as absolute population risk estimates. Accordingly, model performance should be interpreted primarily in terms of discrimination rather than absolute risk estimation. Decision curve analysis was interpreted within the context of the study sampling design. Pathology-based criteria were also applied during cohort definition to exclude small-cell lung cancer and advanced disease, which may introduce potential selection bias and should be considered when interpreting the preoperative applicability of the model. In addition, the absence of separate training and independent validation cohorts represents a methodological limitation, although internal validation procedures were applied to mitigate the risk of overfitting. However, the use of a uniform imaging protocol and a single PET/CT system strengthens the internal consistency of the radiomic analysis. Furthermore, the simultaneous evaluation of intratumoral and peritumoral regions constitutes a methodological strength, as it allows assessment of both tumor-intrinsic characteristics and microenvironment-related imaging features. Also, tumor segmentation was performed by a single experienced reader, and formal interobserver variability or feature robustness analysis (e.g., intraclass correlation coefficient) was not conducted. This may affect the reproducibility of some radiomic features and should be considered when interpreting the results. External validation in multicenter, prospective cohorts will be essential to confirm the robustness and clinical utility of the proposed models. Such an approach may facilitate personalized surgical strategies and contribute to precision medicine in early-stage lung cancer.

## 5. Conclusions

In conclusion, this study demonstrates that STAS, an aggressive and clinically meaningful invasion pattern, can be predicted preoperatively through the integration of ^18^F-FDG PET/CT–based radiomic features derived from both intratumoral and peritumoral regions, together with clinical data.

The developed clinicoradiomic model extends beyond anatomy-based assessment and offers a noninvasive approach to capturing tumor biological behavior at an earlier stage. This strategy may support biology-driven risk stratification and surgical planning in lung cancer and provides a strong rationale for future prospective and multicenter investigations focusing on STAS-oriented personalized management.

## Figures and Tables

**Table 1:** Definitions of Conventional, Advanced Metabolic, and Radiomic Features.

Index	Matrix	Parameter
Conventional indices		SUV_min_, SUV_mean_, SUV_max_, SUV_peak_, SUV_Std_
Volumetric indices		MTV, TLG
	GLCM	Homogeneity, Energy, Contrast, Correlation, Entropy, Dissimilarity
Radiomic Texture features	NGLDM	Coarseness, Contrast, Busyness
	GLRLM	SRE, LRE, LGRE, HGRE, SRLGE, SRHGE, LRLGE, LRHGE, GLNU, RLNU, RP
	GLZLM	SZE, LZE, LGZE, HGZE, SZLGE, SZHGE, LZLGE, LZHGE, GLNU, ZLNU, ZP
Shape features		Sphericity, Surface, Compacity

SUV, standard uptake value; TLG, total lesion glycolysis; MTV, metabolic tumor volume; GLCM, gray-level co-occurrence matrix; NGLDM, neighborhood gray-level difference matrix; GLRLM, gray-level run-length matrix; GLZLM, gray-level zone-length matrix; SRE, short-run emphasis; LRE, long-run emphasis; LGRE, low gray-level run emphasis; HGRE, high gray-level run emphasis, SRLGE, short-run low gray-level emphasis; SRGHE, short-run high gray-level emphasis; LRLGE, long-run row gray-level emphasis; LRHGE, long-run high gray-level emphasis; GLNU, gray-level non-uniformity; RLNU, run-length non-uniformity; RP, run percentage; SZE, short-zone emphasis; LZE, long-zone emphasis; LGZE, low gray-level zone emphasis; HGZE, high gray-level zone emphasis; SZLGE, short-zone low gray-level emphasis; SZHGE, short-zone high gray-level emphasis; LZLGE, long-zone low gray-level emphasis; LZHGE, long-zone high gray-level emphasis; ZLNU, zone-length non-uniformity; ZP, zone percentage.

**Table 2:** Radiomic-Only Model for Preoperative Prediction of STAS.

Predictor	Odds Ratio (OR)	%95 CI	*p*-Value
SUVmin_tumor	0.624	0.413–0.942	0.025
SUVskewness_tumor	0.174	0.042–0.723	0.016
Peritumoral NGLDM_Coarseness	7.61 × 10^16^	Very wide CI	0.399
GLRLM_LRLGE_tumor	-	-	>0.05
GLZLM_LZLGE_tumor	-	-	>0.05
Intratumoral NGLDM_Coarseness	-	-	>0.05

Variables were selected using LASSO-based feature selection. Predictors not reaching statistical significance in multivariable analysis are shown for transparency. The peritumoral NGLDM_Coarseness variable has a very small numeric scale; therefore, odds ratios expressed per 1-unit increase may appear extremely large and are accompanied by very wide confidence intervals, indicating numerical instability. This feature was retained as an exploratory covariate and should be interpreted with caution.

**Table 3:** Combined Clinicoradiomic Model for Preoperative Prediction of STAS.

Predictor	Odds Ratio (OR)	%95 CI	*p*-Value
Eosinophil count (per cell/mm^3^)	0.993	0.987–0.999	0.024
SUVmin_tumor	0.624	0.413–0.942	0.025
SUVskewness_tumor	0.174	0.042–0.723	0.016
Peritumoral NGLDM_Coarseness	7.61 × 10^16^	Very wide CI	0.399
GLRLM_LRLGE_tumor	-	-	>0.05
GLZLM_LZLGE_tumor	-	-	>0.05
Intratumoral NGLDM_Coarseness	-	-	>0.05

Variables were selected using LASSO-based feature selection. Predictors not reaching statistical significance in multivariable analysis are shown for transparency.

## Data Availability

The data supporting the findings of this study are not publicly available due to patient privacy and ethical restrictions. De-identified data may be made available from the corresponding author upon reasonable request and with institutional approval.

## Supplementary Materials

The following supporting information can be downloaded at: https://www.mdpi.com/article/10.3390/diagnostics16050784/s1, Table S1: Final Combined Clinicoradiomic Model (TRIPOD Reporting). Table S2: Final Radiomic Model (TRIPOD Reporting).

## Abbreviations

STAS, spread through air spaces; PET/CT, positron emission tomography/computed tomography; ^18^F-FDG, fluorine-18 fluorodeoxyglucose; SUV, standardized uptake value; SUVmin, minimum standardized uptake value; MTV, metabolic tumor volume. TLG, total lesion glycolysis; GLCM, gray-level co-occurrence matrix; GLRLM, gray-level run-length matrix; NGLDM, neighborhood gray-level difference matrix; GLZLM, gray-level zone-length matrix; ROC, receiver operating characteristic; AUC, area under the curve; LDH, lactate dehydrogenase; CRP, C-reactive protein; NLR, neutrophil-to-lymphocyte ratio.
