# Supplementary material for: Preoperative Prediction of Spread Through Air Spaces in Lung Cancer Using 18F-FDG PET–Based Radiomics and Peritumoral Microenvironment Features
Source: Diagnostics (Basel). 2026 Mar 5;16(5):784. doi: 10.3390/diagnostics16050784 (PMC12984867; doi:10.3390/diagnostics16050784)
Supplement: Supplementary file 1 [file diagnostics-16-00784-s001.zip › diagnostics-4137794-supplementary.pdf]

Table S1. Final Combined Clinicoradiomic Model (TRIPOD Reporting).

Predictor	B (β)	Odds Ratio (OR)	95% CI	p-value
**Intercept**	3.026	—	—	—
Eosinophil count (per cell/mm^3)	−0.00717	0.993	0.987–0.999	0.024
Neutrophil count (per cell/mm^3)	−0.00018	0.9998	—	>0.05
SUVmin_tumor	−0.472	0.624	0.413–0.942	0.025
SUVskewness_tumor	−1.746	0.174	0.042–0.723	0.016
Peritumoral NGLDM_Coarseness	38.871	7.61×10^{16}	Very wide	0.399
GLRLM_LRLGE_tumor	0.128	1.14	—	>0.05
Intratumoral NGLDM_Coarseness	14.396	1.79×10^{6}	—	>0.05
GLZLM_LZLGE_tumor	0.00019	1.00	—	>0.05

The peritumoral NGLDM_Coarseness variable has a very small numeric scale; therefore, odds ratios expressed per 1-unit increase may appear extremely large and are accompanied by very wide confidence intervals, indicating numerical instability. This feature was retained as an exploratory covariate and should be interpreted with caution.

The final combined clinicoradiomic model was defined as:
logit(P(STAS)) = 3.026
− 0.00717 × eosinophil count
− 0.00018 × neutrophil count
− 0.472 × SUVmin_tumor
− 1.746 × SUVskewness_tumor

- 38.871 × peritumoral NGLDM_Coarseness
- 0.128 × GLRLM_LRLGE_tumor
- 14.396 × intratumoral NGLDM_Coarseness
- 0.00019 × GLZLM_LZLGE_tumor.

The predicted probability of STAS was calculated as
P = 1 / (1 + e^(−logit)).

The final combined clinicoradiomic model was defined as:
logit(P(STAS)) = 3.026
− 0.00717 × eosinophil count
− 0.00018 × neutrophil count
− 0.472 × SUVmin_tumor
− 1.746 × SUVskewness_tumor

- 38.871 × peritumoral NGLDM_Coarseness
- 0.128 × GLRLM_LRLGE_tumor
- 14.396 × intratumoral NGLDM_Coarseness
- 0.00019 × GLZLM_LZLGE_tumor.

The predicted probability of STAS was calculated as
P = 1 / (1 + e^(−logit)).

Table S2. Final Radiomic Model (TRIPOD Reporting).

Predictor	B (β)	Odds Ratio	95% CI	p-value
Intercept	1.842	—		
SUVmin_tumor	−0.472	0.624	0.413–0.942	0.025
SUVskewness_tumor	−1.746	0.174	0.042–0.723	0.016

The peritumoral NGLDM_Coarseness variable has a very small numeric scale; therefore, odds ratios expressed per 1-unit increase may appear extremely large and are accompanied by very wide confidence intervals, indicating numerical instability. This feature was retained as an exploratory covariate and should be interpreted with caution.
